# Cardiovascular health outcomes of mobbing at work: results of the population-based, five-year follow-up of the Gutenberg health study

**DOI:** 10.1186/s12995-020-00266-z

**Published:** 2020-06-12

**Authors:** Karla Romero Starke, Janice Hegewald, Andreas Schulz, Susan Garthus-Niegel, Matthias Nübling, Philipp S. Wild, Natalie Arnold, Ute Latza, Sylvia Jankowiak, Falk Liebers, Karin Rossnagel, Merle Riechmann-Wolf, Stephan Letzel, Manfred Beutel, Norbert Pfeiffer, Karl Lackner, Thomas Münzel, Andreas Seidler

**Affiliations:** 1 Institute and Policlinic of Occupational and Social Medicine (IPAS), Faculty of Medicine Carl Gustav Carus, Technische Universität Dresden, Fetscherstrasse 74, 01307 Dresden, Germany; 2 Institute for Sociology, Technische Universität Chemnitz, Chemnitz, Germany; 3 Preventive Cardiology and Preventive Medicine, Center for Cardiology, University Medical Center of the Johannes Gutenberg University of Mainz, Mainz, Germany; 4 FFAW: The Freiburg Research Centre for Occupational and Social Medicine, Freiburg, Germany; 5 DZHK (German Center for Cardiovascular Research), Partner Site Rhein-Main, Mainz, Germany; 6 Center of Thrombosis and Hemostatis (CTH), University Medical Center Mainz, Mainz, Germany; 7 Division Work and Health, Federal Institute for Occupational Safety and Health, BAuA, Berlin, Germany; 8 Institute for Teachers’ Health, University Medical Center of the Johannes Gutenberg University of Mainz, Mainz, Germany; 9 Institute of Occupational, Social, and Environmental Medicine, University Medical Center of the Johannes Gutenberg University of Mainz, Mainz, Germany; 10 Department of Psychosomatic Medicine and Psychotherapy, University Medical Center Mainz, Johannes Gutenberg University Mainz, Mainz, Germany; 11 Deparment of Ophtalmology, University Medical Center Mainz, Johannes Gutenberg University Mainz, Mainz, Germany; 12 Institute for Clinical Chemistry and Laboratory Medicine, University Medical Center Mainz, Johannes Gutenberg University Mainz, Mainz, Germany

**Keywords:** Workplace mobbing, Bullying, Cardiovascular diseases, Hypertension, Arterial stiffness, Occupational health

## Abstract

**Background:**

The aim of this study was to determine if there is an increased risk of incident cardiovascular disease (CVD) resulting from workplace mobbing measured with two mobbing instruments in the Gutenberg Health Study.

**Methods:**

In this prospective study, we examined working persons younger than 65 years for the presence of mobbing at baseline and at a 5-year follow-up using a single-item and a 5-item instrument. We used multivariate models to investigate the association between mobbing and incident CVD, hypertension, and change in arterial stiffness and further stratified the models by sex.

**Results:**

After adjustment for confounders, mobbed workers appeared to have a higher risk of incident CVD than those not mobbed (single-item HR = 1.28, 95% CI 0.73–2.24; 5-item HR = 1.57, 95% CI 0.96–2.54). With the 5-item instrument, men who reported mobbing had a higher risk of incident CVD (HR = 1.77, 95% CI 1.01–3.09), while no association was observed for women (HR = 1.05, 95% CI 0.38–2.91). There was no difference in risks between men and women with the single-item instrument. No association between mobbing and incident hypertension and arterial stiffness was seen.

**Conclusions:**

Our results show an indication of an increased risk of incident CVD for those mobbed at baseline when using the whole study population. Differences in risks between men and women when using the five-item instrument may be due to the instrument itself. Still, it is essential to detect or prevent workplace mobbing, and if present, to apply an intervention to halt it in order to minimize its adverse effects on CVD.

## Background

Mobbing has generated considerable research interest over the previous decades [1, 2] because it is an important psychological stress factor at work. Einarsen et al. (2003), [3] define mobbing as “harassing, offending, socially excluding someone or negatively affecting someone’s work tasks. In order for the label mobbing to be applied ( …) it has to occur repeatedly and regularly over a period of time.” Leymann first used the word “mobbing” as an alternative to “bullying”, as he rather associated bullying with physical aggression and threat [4]. However, the terms mobbing [4, 5], bullying [6], and harassment [7] have been all used in a similar context, and thus the terms have also been used interchangeably [6, 8, 9]. In addition, there is a geographical and cultural component of the terms used. In Germany, Italy, and Sweden, the term “mobbing” is preferred, while in the United Kingdom, this phenomenon is called “workplace bullying” [8]. In this paper, the terms mobbing and workplace bullying are thus used as synonyms.

Mobbing is not only an interpersonal issue, but an organizational dynamic that affects all who are exposed, including bystanders, witnessing colleagues and the workplace as a whole [10–12]. Recent studies have shown that mobbing is one of the psychosocial factors most closely related to health related outcomes such as general health, depression, and burnout [1, 5, 13, 14]. There have been few prospective studies investigating the effect of mobbing on cardiovascular (CVD) outcomes [1, 15], and more are needed. To our knowledge, no studies have investigated the effect of mobbing on sub-clinical CVD outcomes and potential differences in health effects by sex.

We aimed to assess the impact of mobbing at work on CVD outcomes and to explore the association between mobbing and the sub-clinical CVD outcomes hypertension and arterial stiffness (AS). Furthermore, we aimed to investigate if the association is modified by sex. We evaluated whether these associations were dependent on the mobbing assessment instrument applied. For this, we used baseline and five-year follow-up data from a large cohort.

## Methods

### Design and participants

The GHS is a population-based, prospective, single-center cohort study in the Rhine-Main region in Germany [16–18]. Its primary aim is to evaluate and improve cardiovascular risk stratification, and for this purpose, several environmental, psychosocial, and lifestyle factors were investigated. The random sample, drawn from the registry offices in the city of Mainz and the district of Mainz-Bingen, was stratified for sex, residence (urban vs rural), and for equal strata for decades of age. Individuals between the ages of 35 and 74 years were enrolled after obtaining written informed consent. Participants were excluded if they had insufficient knowledge of the German language or if they declared a physical or psychological inability to participate in the examinations at the study center. Between April 2007 and April 2012, 15,010 participants were recruited with a response of 60% [18]. The study was approved by the local ethics committee and by the local and federal data safety commissioners (#837.020.07(5555)).

**Fig. 1:**
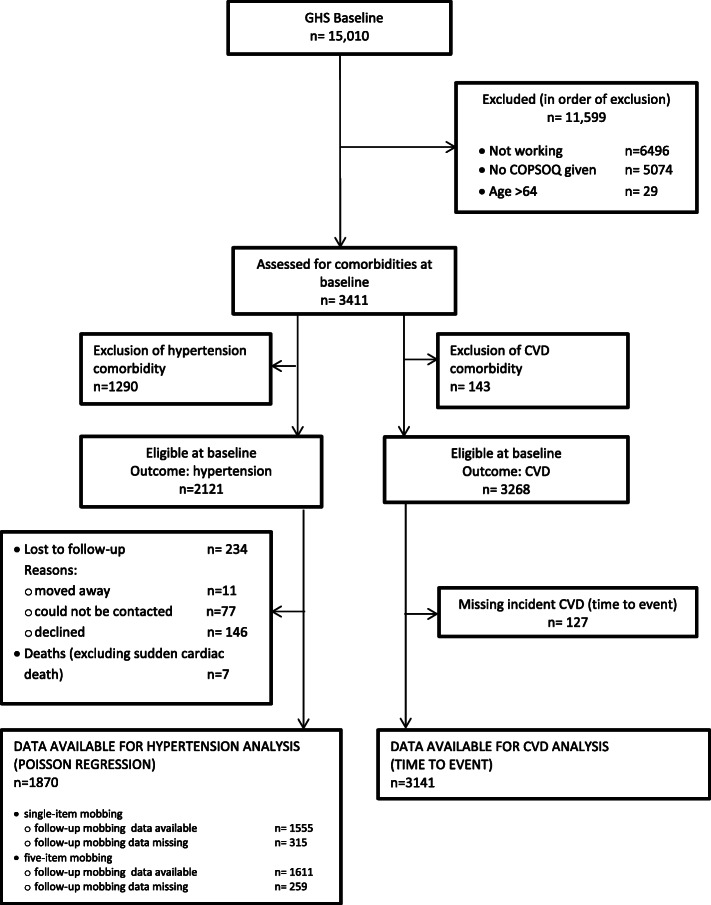
GHS participant process flow

For this study, of the initial 15,010 participants, we excluded those not working at baseline (*N* = 6496), those who did not receive the COPSOQ assessment (given randomly to approximately half of the working participants), and those who were older than 64 years. We also excluded those with prevalent CVD (for the CVD analysis) or hypertension (for the hypertension analysis) at baseline. At baseline, there were 3268 eligible participants for the CVD analysis and 2121 for the hypertension analysis. After a five-year follow-up, 3141 and 1870 participants remained for the CVD and hypertension analysis, respectively. The detailed process flow is shown in Fig. 1.

### Measures

#### Mobbing measures

For this study, both a single-item and a five-item mobbing instrument were included in the German version of the Copenhagen Psychosocial Questionnaire (COPSOQ) [19]. The COPSOQ was given to working participants through computer-assisted personal interviews at baseline and at the follow-up. The single-item mobbing instrument asked: “How often do you feel unjustly criticized, bullied, or shown up in front of others by your colleagues and your superior?”. Answer categories were “always”, “often”, “sometimes”, “seldom”, and “never/hardly ever”. For our analysis, mobbing was dichotomized in the following manner: “seldom and never/hardly ever” (no mobbing) vs “sometimes/often/always” (mobbing).

The five-item mobbing scale was developed from the LIPT31 “Leymann Inventory of Psychological Terror” [4] to achieve maximum sensitivity. More details can be found in Garthus-Niegel et al. [20]. The five-item instrument asked:

1. Do you get intentionally interrupted during oral contributions?
2. Does it happen that you receive no response/ reaction, when you want to speak to someone?
3. Do you get blamed for others’ mistakes or general operational problems?
4. Were important influential or working areas taken away from you?
5. Did you receive unpleasant sexual offers or did you get sexually harassed?

If an item was answered with “yes” the respondents were asked about the frequency: daily/almost daily, at least one time per week, at least one time per month, and less than one time per month. A dichotomized mobbing construct for the five-item scale was created: “less than once a week in all of the five constructs” (no mobbing), vs “at least once a week in at least one out of the five constructs” (mobbing).

#### Cardiovascular outcomes

Our primary outcome of interest was incident diagnoses of ischemic diseases, defined as first acute myocardial infarct (ICD-10: I21), cerebral infarction/ischemic stroke (ICD-10: I63), atrial fibrillation (ICD I48), peripheral artery disease (ICD-10: I73.9), coronary artery disease (ICD-10: I25.10), chronic heart failure (ICD-10: I50, I11.0, I13.0, I13.2), and sudden cardiac death (ICD-10: I46). All the above were grouped as “incident case of CVD” and analyzed together. Hospital and doctors’ records, as well as death certificates were used to identify CVD events and to define the date of first occurrence after baseline. A team of experts validated and confirmed each CVD event.

We assessed the sub-clinical CVD health outcomes of incident hypertension (ICD-10: I10) and AS, both measured at the baseline and follow-up visits. Hypertension was defined as having a mean systolic blood pressure of ≥140 mmHg or a mean diastolic blood pressure of ≥90 mmHg (in the 2nd and 3rd standardized measurement after 8 and 11 min of rest), or self-reported use of anti-hypertensive drugs. AS was measured by digital photoplethysmography using a Pulse Trace PCA2 device (Micro Medical Limited/Carefusion). The AS index was calculated by the height (in meters) divided by the difference between early systolic and second diastolic peak (in seconds). A high-flow velocity expresses inelastic arterial blood vessels [21].

#### Covariates

We considered sex, age, socioeconomic status (SES), smoking, alcohol, waist-to-height ratio (WHtR), physical activity, the presence of anxiety, depression, and type D personality at baseline as potential confounders for the association between mobbing and cardiovascular outcomes. SES was assessed using an index score constructed from information on school education, professional education, occupational position, and salary [22, 23]. A binary variable differentiated between nonsmokers and current/occasional smokers for the past 6 months. Alcohol intake was categorized per the “tolerable upper alcohol intake levels” (TOAM) [24]. We used the WHtR measure as it has been found to be a better predictor for cardiovascular outcomes than body mass index [25]. Physical activity was evaluated using the Short Questionnaire to Assess Health-Enhancing (SQUASH) Physical Activity questionnaire [26]. The presence of depression was measured with the Patient Health Questionnaire (PHQ-9) using a cutoff score of ≥10, while presence of anxiety disorder was determined by a cutoff score ≥ 3 on the 2-item version of the Generalized Anxiety Disorder Scale (GAD-7). Distressed (or Type D) personality, characterized by a tendency towards negative affectivity and social inhibition was assessed with the German version of the Type D scale (DS14), and was defined by a cutoff score of ≥10 on the negative affectivity and social inhibition scales [27, 28].

### Statistical analysis

To test the association of mobbing at baseline and incident CVD, we estimated hazard ratios (HR) using Cox regression using deaths due to causes other than CVD as censored observations. The time to event was defined by the number of years after baseline participation to first occurrence of CVD events. Participants who dropped out due to reasons not related to CVD were censored. For the association between mobbing at baseline and incident hypertension, we used robust Poisson regression [29]. We used multivariate linear regression models using all available data to evaluate the effect of mobbing on the change in AS index from baseline to follow-up.

Five different adjustment sets which were defined a priori were used to study possible confounding effects of the covariates of interest on the association between mobbing and CVD outcomes. We further stratified for gender to examine possible sex-related differences in disease risks. All analyses were conducted using R version 3.6.0 (2019).

### Sensitivity analysis

In a sensitivity analysis, we considered the effect of chronic mobbing on hypertension and AS by combining mobbing at baseline and follow-up, and created a categorical variable for no mobbing at either assessment (reference), mobbing at baseline only, mobbing at follow-up only (incident mobbing), and recurrent mobbing (mobbing at baseline and at follow-up).

Additionally, the risk for incident CVD was investigated for each of the items comprising the five-item instrument for all participants and separately for men and women.

## Results

### Sample characteristics

The baseline sample characteristics for the participants included in the analysis of incident CVD are presented in Table 1. The average age of the sample was 47.6 years and 46% were women. For the single-item construct, 15.4% of the participants reported experiencing workplace mobbing at baseline, and both men and women had similar mobbing prevalences (15.0 and 15.9%, respectively). However, for the five-item mobbing construct, 19.9% reported mobbing at baseline, with more men than women reporting being mobbed at baseline (21.3 and 18.2%, respectively). Those who reported mobbing at baseline had slightly higher WHtR, were more likely to suffer from depression, to have a type D personality, and to suffer from anxiety than those who did not report mobbing. Baseline characteristics for the participants included in the hypertension analysis are available in Supplementary Table 1.

**Table 1:** Baseline characteristics for the CVD analysis, *N* = 3268

Characteristics	Single-item mobbing at baseline		Five-item mobbing at baseline	
	No mobbing (***N*** = 2764)	Mobbing (***N*** = 504)	No mobbing (***N*** = 2619)	Mobbing (***N*** = 649)
**Sex**				
Women	1270 (45.9%)	240 (47.6%)	1235 (47.2%)	275 (42.4%)
Men	1494 (54.1%)	264 (52.4%)	1384 (52.8%)	374 (57.6%)
**Age** Average (SD)	47.7 (7.5)	47.2 (7.3)	47.5 (7.4)	48.2 (7.6)
**SES** Average (SD)	14.5 (4.1)	13.1 (4.1)	14.3 (4.1)	14.1 (4.3)
**WHtR Average (SD)**	0.53 (0.07)	0.54 (0.08)	0.53 (0.07)	0.54 (0.08)
**Physical activity score Average (SD)**	8.3 (3.6)	9.0 (4.4)	8.3 (3.6)	8.9 (4.2)
**Alcohol intake**				
Below limit	2173 (78.6%)	407 (80.8%)	2084 (79.6%)	496 (76.4%)
Above limit	591 (21.4%)	97 (19.2%)	535 (20.4%)	153 (23.6%)
**Smoking status**				
Current/occasional	629 (22.8%)	126 (25.0%)	590 (22.5%)	165 (25.4%)
Nonsmoker	2135 (77.2%)	378 (75.0%)	2029 (77.5%)	484 (74.6%)
**Depression (PHQ-9 ≥ 10)**				
Yes	166 (6.0%)	88 (17.5%)	154 (5.9%)	100 (15.5%)
No	2594 (94.0%)	416 (82.5%)	2462 (94.1%)	547 (84.5%)
**Type D Personality**				
Yes	598 (21.7%)	178 (35.4%)	586 (22.4%)	190 (29.5%)
No	2160 (78.3%)	325 (64.6%)	2030 (77.6%)	455 (70.5%)
**Anxiety**				
Yes	288 (10.4%)	126 (25.0%)	272 (10.4%)	142 (22.0%)
No	2470 (89.6%)	377 (75.0%)	2342 (89.6%)	504 (78.0%)

*SES* socioeconomic status

*WHtR* Waist to height ratio

*PHQ* Patient Health Questionnaire

Of the participants who completed both the baseline and follow-up mobbing instruments, 5 and 7% reported recurrent mobbing using the single-item and five-item construct, respectively. The main reason for not filling out the COPSOQ questionnaire at follow-up was being retired (66%).

### Incident CVD

Altogether, 80 incident CVD events and 11 competing events (non-CVD deaths) occurred among the subsample during the follow-up, corresponding to 15,229 person-years. The unadjusted incidence rate ratio for the subsample was 1.30 (95% CI 0.69–2.31) for the single-item construct and 1.78 (95% CI 1.05–2.91) for the five-item construct (Table 2).

**Table 2:** Crude associations of mobbing with incidence of CVD (time to event)

	Subjected to mobbing at baseline	N	Number of events	Censored events	Person-years	Incidence rate (per 1000 person-years)	Unadjusted incidence rate ratio (95% CI)
**Single-item construct**							
All	No mobbing	2665	65	2600	12,934	5.03	1
	Mobbing	476	15	461	2295	6.54	1.30 (0.69–2.31)
Men	No mobbing	1434	47	1387	6916	6.80	1
	Mobbing	252	11	241	1218	9.03	1.33 (0.62–2.60)
Women	No mobbing	1231	18	1213	6018	2.99	1
	Mobbing	224	4	220	1077	3.71	1.24 (0.31–3.77)
**Five-item construct**							
All	No mobbing	2523	56	2476	12,268	4.56	1
	Mobbing	618	24	594	2961	8.10	1.78 (1.05–2.91)
Men	No mobbing	1329	39	1290	6436	6.06	1
	Mobbing	357	19	338	1698	11.19	1.85 (1.01–3.27)
Women	No mobbing	1194	17	1177	5833	2.91	1
	Mobbing	261	5	256	1263	3.96	1.36 (0.39–3.84)

After adjustment for age, sex, SES, smoking, WHtR, physical activity, and alcohol consumption, there was an increased risk of incident CVD for those reporting mobbing at baseline using the single-item mobbing construct (HR = 1.28; 95% CI: 0.73–2.24) (Table 3). When using the five-item construct, participants who reported mobbing at baseline were at a higher risk for incident CVD than those who reported no mobbing at baseline (HR = 1.57; 95% CI 0.96–2.54). Further adjustment for depression, anxiety, and type D personality yielded a lower association for both mobbing constructs (single-item: HR = 1.11, 95% CI 0.64–1.94; five-item: HR = 1.45, 95% CI 0.89–2.38).

**Table 3:** Associations of mobbing with incidence of CVD (time to event)

		Hazards Ratio (HR)^a^ (95% CI)	HR^b^ (95%CI)	HR^c^ (95% CI)	HR^d^ (95%CI)
**Single-item construct**					
All	No mobbing	1.00	1.00	1.00	1.00
(*n* = 3141) 80 events, 11 competing events	Mobbing	1.31 (0.74–2.29)	1.32 (0.75–2.31)	1.28 (0.73–2.24)	1.11 (0.64–1.94)
Men	No mobbing	1.00	1.00	1.00	1.00
(*n* = 1686) 58 events, 7 competing events	Mobbing	1.31 (0.68–2.52)	1.32 (0.69–2.53)	1.28 (0.68–2.41)	1.16 (0.61–2.21)
Women	No mobbing	1.00	1.00	1.00	1.00
(*n* = 1455) 22 events, 4 competing events	Mobbing	1.27 (0.41–3.85)	1.31 (0.42–4.06)	1.20 (0.39–3.69)	0.96 (0.32–2.83)
**Five-item construct**					
All	No mobbing	1.00	1.00	1.00	1.00
(n = 3141) 80 events, 11 competing events	Mobbing	1.61 (0.99–2.60)	1.61 (0.99–2.61)	1.57 (0.96–2.54)	1.45 (0.89–2.38)
Men (n = 1686) 58 events, 7 competing events	No mobbing	1.00	1.00	1.00	1.00
	Mobbing	1.77 (1.02–3.08)	1.80 (1.03–3.14)	1.77 (1.01–3.09)	1.70 (0.97–3.00)
Women	No mobbing	1.00	1.00	1.00	1.00
(n = 1455) 22 events, 4 competing events	Mobbing	1.15 (0.41–3.19)	1.14 (0.41–3.13)	1.05 (0.38–2.91)	0.82 (0.29–2.32)

^a^adjusted by age, sex, socioeconomic status (SES)

^b^adjusted by age, sex, SES, smoking

^c^adjusted by age, sex, SES, smoking, physical activity score, alcohol, waist to height ratio (WHtR)

^d^adjusted by age, sex, SES, smoking, physical activity score, alcohol, WHtR, depression, anxiety, type D personality

When using the single-item instrument, and after adjusting for age, SES, smoking, and WHtR, there was a higher risk for both men and women who were mobbed. For the five-item instrument, men who were mobbed at baseline had a 77% increased risk compared to men who were not mobbed (HR = 1.77, 95% CI 1.01–3.09). However, this association was not observed for women (HR = 1.05, 95% CI 0.38–2.91). The sensitivity analysis (Supplementary Table 2) showed that more men (9.4%) than women (5.2%) reported being blamed for others’ mistakes or for operational problems. Six people, all of them women, reported being sexually harassed. All other items were similarly reported for men and women. The risk for incident CVD was generally higher for men than for women in each item in the five-item construct.

### Sub-clinical cardiovascular disease: hypertension and AS

Throughout the follow-up there were 332 incident cases of hypertension. There was no association between mobbing at baseline using both item constructs and incident hypertension (Table 4) or change in AS (Supplementary Table 3). No association was observed between mobbing at baseline and hypertension or AS for men or women (results not shown). Sensitivity analyses also showed no association between mobbing at baseline, incident, or recurrent mobbing and incident hypertension or change in AS (Supplementary Tables 4 and 5).

**Table 4:** Associations of mobbing with incidence of hypertension, *n* = 1870

Mobbing at baseline	N	RR^a^ (95%CI)	RR^b^ (95%CI)	RR^c^ (95%CI)	RR^d^ (95%CI)
Single-item construct					
No mobbing (ref)	1191	1.00	1.00	1.00	1.00
Mobbing	151	0.97 (0.73–1.27)	0.97 (0.73–1.28)	0.93 (0.70–1.22)	0.95 (0.72–1.25)
Five-item construct					
No mobbing (ref)	1145	1.00	1.00	1.00	1.00
Mobbing	179	1.04 (0.82–1.32)	1.04 (0.82–1.32)	0.99 (0.78–1.25)	1.00 (0.80–1.27)

^a^adjusted by age, sex, socioeconomic status (SES)

^b^adjusted by age, sex, SES, smoking

^c^adjusted by age, sex, SES, smoking, physical activity score, alcohol, waist to height ratio (WHtR)

^d^adjusted by age, sex, SES, smoking, physical activity score, alcohol, WHtR, depression, anxiety, type D personality

## Discussion

This population-based longitudinal study investigated the effects of mobbing on incident CVD and pre-clinical CVD outcomes. After adjusting for age, sex, SES, smoking, physical activity, and alcohol, participants exposed to mobbing at baseline had a 28–57% higher risk of incident CVD than those who did not report mobbing, depending on the mobbing instrument used. Using the single-mobbing instrument resulted in a 28% higher risk for those mobbed at baseline, although the association was not significant; the five-item instrument resulted in a 57% higher risk for those mobbed at baseline. The association was attenuated and no longer significant for the five-item instrument after additional adjustments for depression, anxiety, and type D personality. Depression and anxiety may be risk factors for CVD, but they also may be intermediate variables on the causal pathway between mobbing and CVD: mobbing can generate depressiveness and with that, a change of health behaviors. An over-adjustment for intermediate variables may result in over-adjustment biasing to the null [30]. Indeed, several prospective studies have seen an increase in depression, anxiety and mental health problems on people who are mobbed [1, 31–33] and these psychological conditions are known risk factors for CVD [16, 34, 35]. Neither instrument showed an association between mobbing and the sub-clinical CVD outcomes hypertension and AS.

When the five-item instrument was used, there was a particularly strong association between mobbing and CVD- but only in men, and not in women. For the interpretation of these results, it should be noted that the five-item instrument was formed on the basis of a study population consisting predominantly of men [36], and therefore the depiction of mobbing by the five-item instrument in women may be limited. In this study, more men reported being mobbed at baseline than women with the five-item instrument. Comparisons in the mobbing experiences in women and men have shown differences between both sexes, and that women are predominantly affected by attacks in the social context (through ostracism or social isolation, insults, or teasing), while men are affected by attacks in the professional context (through unfair work criticism or withdrawal of work tasks) [37]. This was confirmed by our sensitivity analysis, which showed that women were less affected by “getting blamed for others’ mistakes” than men (similar to the results from the baseline study [20]). Additionally, there were only six people (all women) who reported having been sexually harassed, which may indicate an unsuitability of this question with regards to mobbing. Garthus-Niegel and authors previously reported that the sexual harassment item had a low-factor loading [20]. On the other hand, when using the single-item instrument, men and women reported similar mobbing prevalences and risks for incident CVD (albeit not significant). It is possible that the single-item instrument may offer a more general mobbing definition for both sexes, “without pinpointing concrete acts, which allows for measuring a wide array of mobbing strategies” [20]. Yet, the mobbing risk effects found when using the single-item instrument could not mirror those observed when using the five-item instrument in men. The five-item instrument may be a more precise instrument for men, and the higher risk of incident CVD due to mobbing observed in men, and not in women, when using the five-item instrument is likely to be an artifact of the instrument itself. Sex-specific differences between the mobbing instruments were also found in the baseline study while investigating other outcomes [20].

### Comparison to other studies

In this study, the prevalence of mobbing at baseline varied depending on the mobbing instrument used. It has been found that using Leymann’s definition of mobbing results in a higher reported prevalence of mobbing than using a stricter criterion [38], and that there is a general difference in mobbing prevalence depending on the measurement method used [39]. A recent study in Germany, which used a similar measure of mobbing to the five-item instrument, reported a mobbing prevalence of 17%, similar to our results [40]. Regional differences may also explain the differences in mobbing prevalences: studies in Norway, Finland, England, and the US, have reported mobbing prevalences ranging from 5 to 41% [1, 6, 41, 42].

Depending on the instrument used, 58–66% of workers who were mobbed at baseline did not report being mobbed at the follow-up, similar to what Kivimäki and colleagues found [1]. As Kivimäki noted, this is an indication that mobbing may be short-lived, perhaps due to personal coping strategies, interventions, or changes in the work environment.

Kivimäki and colleagues [1] also reported an increased risk of CVD due to mobbing. More recently, a multi-center prospective study from Sweden and Denmark using verified CVD outcomes found that workers who were mobbed had a 59% higher risk of incident CVD than those who were not mobbed at work [15].

### Strengths and limitations

A main strength of this study is its prospective nature. Because participants were prospectively followed up, a potential healthy worker effect, in which participants who are sicker than the rest of the workers leave the workplace, was minimized. Furthermore, CVD outcomes were based on medical records and death certificates, and hypertension and AS were measured in clinics, which ensured non-biased outcome reporting. Loss to follow-up was up to 11% for the hypertension and arterial thickness analysis, acceptable for prospective cohort studies. Relevant confounders were also taken into account in the analyses.

The reported results should be considered in light of their limitations. The five-item instrument may not be appropriate for women, as it was developed using a population sample consisting of a majority of men. In addition, there could be a general underreporting of mobbing for men and women. If a significant proportion of participants underreported mobbing, the association between mobbing and the outcomes of interest may have been underestimated. Persons who declared themselves psychologically unable to participate in the examination were not included in this study, which might result in a bias. If people with severe mobbing-related psychological problems were underrepresented, the effect on the studied risk cannot be determined. This study was done in a representative German working population, and results are generalizable to populations with similar ethnic compositions and social structures.

### Implications for prevention or intervention

Our results indicate that measures to prevent or reduce mobbing at work could be useful to promote cardiovascular health. A review on mental health at work indicated that since mobbing is a complex process, it would make sense to intervene through the organizational, group, and individual levels, using both general (i.e. process changes and team-building) and specific measures, such as sensitization to mobbing [43]. At the organizational level, the company should be open to a cooperative environment where employees are valued, provide training to managers, and promote an open and communicative environment with enough available resources to do the job. At the group level, promoting autonomy, team-work, working structures that support cooperation and options to facilitate the solving of problems and conflicts should enable a mobbing-free environment. At the individual level, training programs for coping with stress, providing career opportunities, and providing resources to support the worker are all strategies to prevent mobbing and to promote a healthy workplace [43].

## Conclusions

Our results show an increased risk of incident CVD for those mobbed at baseline using both mobbing instruments. The implications of these findings on individuals and organizations are clear. It is important to detect workplace mobbing early or prevent it altogether in order to minimize its adverse effects on cardiovascular disease.

## Supplementary information

**Additional file 1: Supplementary Table 1.** Baseline characteristics for the hypertension analysis. **Supplementary Table 2.** Sensitivity analysis using the five-item mobbing instrument. **Supplementary Table 3.** Associations of mobbing at baseline with change in arterial stiffness (AS). **Supplementary Table 4.** Associations of incident and recurrent mobbing with incidence of hypertension. **Supplementary Table 5.** Associations of incident and recurring mobbing with change in arterial stiffness (AS).
