# Supplementary material for: Cardiovascular health outcomes of mobbing at work: results of the population-based, five-year follow-up of the Gutenberg health study
Source: J Occup Med Toxicol. 2020 Jun 12;15:15. doi: 10.1186/s12995-020-00266-z (PMC7291638; doi:10.1186/s12995-020-00266-z)
Supplement: Supplementary file 1 — Additional file 1: Supplementary Table 1. Baseline characteristics for the hypertension analysis. Supplementary Table 2. Sensitivity analysis using the five-item mobbing instrument. Supplementary Table 3. Associations of mobbing at baseline with change in arterial stiffness (AS). Supplementary Table 4. Associations of incident and recurrent mobbing with incidence of hypertension. Supplementary Table 5. Associations of incident and recurring mobbing with change in arterial stiffness (AS). [file 12995_2020_266_MOESM1_ESM.docx]

| **Supplementary Table 1.** Baseline characteristics for the hypertension analysis, n=2121 | | | | | |
| --- | --- | --- | --- | --- | --- |
| **Characteristics** | **Single-item mobbing at baseline** | |  | **Five-item mobbing at baseline** | |
|  | **No mobbing**  **(n= 1794)** | **Mobbing**  **(n= 327)** |  | **No mobbing**  **(n=1710 )** | **Mobbing**  **(n= 411)** |
| **Sex** Women | 892 (49.7%) | 164 (50.2%) |  | 880 (51.5%) | 176 (42.8%) |
| Men | 902 (50.3%) | 163 (49.8%) |  | 830 (48.5%) | 235 (57.2%) |
| **Age** Average(SD) | 46.3 (7.2) | 45.6 (7.0) |  | 46.1 (7.1) | 46.5 (7.4) |
| **SES** Average (SD) | 14.6 (4.0) | 13.5 (4.1) |  | 14.5 (4.0) | 14.4 (4.2) |
| **WHtR** | 0.5 (0.1) | 0.53 (0.1) |  | 0.5 (0.1) | 0.5 (0.1) |
| **Physical activity score** | 8.3 (3.7) | 9.1 (4.4) |  | 8.2 (3.6) | 9.2 (4.3) |
| **Alcohol intake** |  |  |  |  |  |
| Below limit | 1443 (80.4%) | 262 (80.1%) |  | 1382 (80.8%) | 323 (78.6%) |
| Above limit | 351 (19.6%) | 65 (19.9%) |  | 328(19.2%) | 88 (21.4%) |
| **Smoking status** |  |  |  |  |  |
| Current/occasional | 386 (24.1%) | 61 (22.7%) |  | 421 (12.4%) | 115 (28.0%) |
| Nonsmoker | 1215 (75.9%) | 208 (77.3%) |  | 1290 (75.4%) | 296 (72.0%) |
| **Depression (PHQ≥10)** |  |  |  |  |  |
| Yes | 101 (5.6%) | 55 (16.8%) |  | 94 (5.5%) | 62 (15.1%) |
| No | 1692 (94.4%) | 272 (83.2%) |  | 1615 (94.5%) | 349 (84.9%) |
| **Type D Personality** |  |  |  |  |  |
| Yes | 388 (21.7%) | 108 (33.0%) |  | 388 (22.7%) | 108 (26.3%) |
| No | 1404 (78.3%) | 219 (67.0%) |  | 1321 (77.3%) | 302 (73.7%) |
| **Anxiety** |  |  |  |  |  |
| Yes | 183 (10.2%) | 77 (23.5%) |  | 177 (10.4%) | 83 (20.2%) |
| No | 1609 (89.8%) | 250 (76.5%) |  | 1530 (89.6%) | 328 (79.8%) |
| SES: socioeconomic status  WHtR: Waist to height ratio  PHQ: Patient Health Questionnaire | | | | | |

| **Supplementary Table 2.** Sensitivity analysis using the five-item mobbing instrument | | | | | | | |
| --- | --- | --- | --- | --- | --- | --- | --- |
| **Item in the 5-item mobbing instrument** | **Mobbing at baseline** | **All,**  **N (%)** | **Men,**  **N (%)** | **Women,**  **N (%)** | **HR* CVD Time to Event (95% CI)** | | |
|  |  |  |  |  | **All** | **Men** | **Women** |
| 1. Do you get intentionally interrupted during oral contributions? | No mobbing | 2744  (87.4%) | 1474  (87.4%) | 1270  (87.4%) | 1.00 | 1.00 | 1.00 |
|  | Mobbing | 397  (12.6%) | 212  (12.6%) | 185  (12.7%) | 1.24  (0.68, 2.27) | 1.46  (0.74, 2.91) | 0.74  (0.22, 2.45) |
| 1. Does it happen that you receive no response/reaction when you want to speak to someone? | No mobbing | 2962  (94.3%) | 1595  (94.6%) | 1367 (94.0%) | 1.00 | 1.00 | 1.00 |
|  | Mobbing | 179  (5.7%) | 91  (5.4%) | 88  (6.0%) | 2.02  (0.95,4.27) | 2.36  (0.99, 5.63) | 1.09  (0.25, 4.74) |
| 1. Do you get blamed for others’ mistakes or general operational problems? | No mobbing | 2907  (92.6%) | 1527  (90.6%) | 1380  (94.8%) | 1.00 | 1.00 | 1.00 |
|  | Mobbing | 234  (7.4%) | 159  (9.4%) | 75  (5.2%) | 1.48  (0.76-2.87) | 1.72  (0.85-3.49) | 0.71  (0.09-5.63) |
| 1. Were important influential or working areas taken away from you? | No mobbing | 3072  (97.8%) | 1648  (97.7%) | 1424  (97.9%) | 1.00 | 1.00 | 1.00 |
|  | Mobbing | 69  (2.2%) | 38  (2.3%) | 31  (2.1%) | 2.22  (0.86-5.77) | 1.75  (0.54-5.69) | 3.37  (0.67-16.9) |
| 1. Did you receive unpleasant sexual offers or did you get sexually harassed? | No mobbing | 3135  (99.2%) | 1686  (100%) | 1449  (99.6%) | 1.00 | 1.00 | 1.00 |
|  | Mobbing | 6  (0.8%) | 0  (0%) | 6  (0.4%) | - | - | - |

| **Supplementary Table 3.** Associations of mobbing at baseline with change in arterial stiffness (AS), n= 1475 | | | | | |
| --- | --- | --- | --- | --- | --- |
|  |  | **ΔAS ***  **(95%CI)** | **ΔAS †**  **(95%CI)** | **ΔAS ‡**  **(95%CI)** | **ΔAS §**  **(95%CI)** |
| Single-item construct |  |  |  |  |  |
| Mobbing at baseline |  | -0.14  (-0.51-0.23) | -0.14  (-0.51-0.23) | -0.16  (-0.53-0.21) | -0.16  (-0.53-0.22) |
| Five-item construct |  |  |  |  |  |
| Mobbing at baseline |  | 0.17  (-0.15-0.49) | 0.17  (-0.15-0.49) | 0.16  (-0.16-0.48) | 0.17  (-0.15-0.50) |
| *adjusted by age, sex, socioeconomic status (SES)  †adjusted by age, sex, SES, smoking  ‡adjusted by age, sex, SES, smoking, physical activity score, alcohol, waist to height ratio (WHtR)  §adjusted by age, sex, SES, smoking, physical activity score, alcohol, WHtR, depression, anxiety, type D personality  ΔAS: AS_5-year follow-up_ - AS_baseline_ | | | | | |

| **Supplementary Table 4**. Associations of incident and recurrent mobbing with incidence of hypertension | | | | |
| --- | --- | --- | --- | --- |
| **Subjected to mobbing** | **Participants** | **RR*** | **RR†** | **RR‡** |
| Single-item construct |  |  |  |  |
| At neither time (ref) | 1191 | 1.00 | 1.00 | 1.00 |
| At baseline only | 151 | 0.94  (0.63-1.40) | 0.88  (0.59-1.31) | 0.86  (0.58-1.28) |
| Incident mobbing | 137 | 1.31  (0.93-1.82) | 1.23  (0.88-1.71) | 1.22  (0.88-1.69) |
| Recurrent mobbing | 76 | 1.24  (0.79-1.96) | 1.14  (0.73-1.79) | 1.18  (0.75-1.84) |
| Five-item construct |  |  |  |  |
| At neither time (ref) | 1145 | 1.00 | 1.00 | 1.00 |
| At baseline only | 179 | 1.25  (0.92-1.71) | 1.23  (0.91-1.67) | 1.24  (0.91-1.68) |
| Incident mobbing | 170 | 0.96  (0.67-1.37) | 0.93  (0.65-1.34) | 0.94  (0.65-1.36) |
| Recurrent mobbing | 117 | 0.75  (0.47-1.21) | 0.75  (0.47-1.20) | 0.71  (0.45-1.13) |
| *adjusted by age and sex  †adjusted by age, sex, socioeconomic status (SES), smoking  ‡adjusted by age, sex, SES, smoking, physical activity score, alcohol, waist to height ratio, depression, anxiety, type D personality | | | | |

| **Supplementary Table 5.** Associations of incident and recurring mobbing with change in arterial stiffness (AS), n= 1475 | | | |
| --- | --- | --- | --- |
| **Subjected to mobbing** | **ΔAS*** | **ΔAS †** | **ΔAS‡** |
| Single-item construct | | | |
| At baseline only | -0.072  (-0.58, 0.44) | -0.09  (-0.60, 0.41) | -0.10  (-0.61, 0.41) |
| Incident mobbing | 0.16  (-0.35, 0.67) | 0.14  (-0.37, 0.65) | 0.13  (-0.38, 0.65) |
| Recurrent mobbing | -0.36  (-1.00,0.28) | -0.41  (-1.05, 0.23) | -0.46  (-1.12, 0.20) |
| Five-item construct | | | |
| At baseline only | -0.06  (-0.53, 0.41) | -0.06  (-0.54, 0.41) | -0.06  (-0.53, 0.42) |
| Incident mobbing | -0.05  (-0.53, 0.43) | -0.06  (-0.54, 0.42) | -0.036  (-0.52, 0.45) |
| Recurrent mobbing | 0.23  (-0.27, 0.73) | 0.22  (-0.28, 0.72) | 0-24  (-0.28, 0.75) |
| *adjusted by age and sex  †adjusted by age, sex, socioeconomic status (SES), smoking  ‡adjusted by age, sex, SES, smoking, physical activity score, alcohol, waist to height ratio, depression, anxiety, type D personality  ΔAS: AS_5-year follow-up_ - AS_baseline_ | | | |
